# Variability in Standardized Letters of Evaluation: A Multi‐Institutional Review of EM Residency Based Versus Non‐Residency Based Faculty Evaluation

**DOI:** 10.1002/aet2.70133

**Published:** 2026-02-16

**Authors:** Katarzyna Gore, Cullen B. Hegarty, Thomas Beardsley, Sara M. Krzyzaniak, Sandra Monteiro, Al'ai Alvarez, Teresa Davis, Melissa Parsons, Aman Pandey, Sharon Bord, Michael Gottlieb, Alexandra Mannix

**Affiliations:** ^1^ Department of Emergency Medicine Rush University Medical Center Chicago Illinois USA; ^2^ Department of Emergency Medicine HealthPartners Institute/Regions Hospital St. Paul Minnesota USA; ^3^ Department of Emergency Medicine University of Florida College of Medicine Jacksonville Florida USA; ^4^ Department of Emergency Medicine Stanford University Stanford California USA; ^5^ Department of Medicine McMaster University Hamilton Ontario Canada; ^6^ Department of Emergency Medicine The Johns Hopkins University School of Medicine Baltimore Maryland USA

## Abstract

**Background:**

The Standardized Letter of Evaluation (SLOE) in Emergency Medicine (EM) was developed by the Council of Residency Directors in Emergency Medicine (CORD) to have a uniform approach to providing training programs with information about applicants in the match process. Recent revisions distinguish what setting letter writers originate (residency based [RB] training program vs. non‐residency based [nRB] site).

**Objectives:**

The goal of this paper was to compare SLOE 2.0 scoring between letter writers from RB versus nRB settings.

**Methods:**

This was a multi‐institutional cross‐sectional study. The study team from five residency programs collected data from SLOEs in their applicant pool from 2022 to 2023 match cycle. Each SLOE was reviewed for training location/SLOE type (RB vs. nRB) and numerical scores for sections A, B, and C (Anticipated Guidance [AG]). The data were not normally distributed, so were analyzed using descriptive and chi‐squared statistics. Data were examined using Spearman's Rho (*⍴*) to evaluate the relationship of Part A and B scores with faculty estimates of AG.

**Results:**

The study analyzed 3687 eSLOEs from 1772 applicants. The majority (*N* = 3526) were from RB faculty with only 161 from nRB faculty. The median scores were similar between groups, but the distribution of Part A and B scores was different between RB and nRB faculty. One exception was A4 (ability to perform common ED procedures) which had similar proportions of scores. There was a positive monotonic relationship between evaluation scores (Part A and B) and faculty estimates for AG, predicting up to 30% variability. Overall eSLOEs from nRB faculty had higher proportions of higher scores.

**Conclusions:**

This study found a significant difference in proportions of scores assigned between RB faculty versus nRB faculty on most items including AG.

## 1 Introduction/Background

The standardized letter of evaluation (SLOE) in Emergency Medicine (EM) is an essential component of a medical student's application to an EM residency. It is often identified by residency program directors (PDs) as the leading metric for determining whether to interview an applicant and also for ranking applicants [1, 2]. Initially developed by the Council of Residency Directors in Emergency Medicine (CORD) in 1995, the SLOE has gone through several revisions over the years to its current form, the electronic SLOE 2.0 (eSLOE 2.0). This summative evaluation incorporating normative grading has proven to be an effective tool and allowed for improved efficiency and inter‐rater reliability over the narrative letter of recommendation [3, 4].

Despite its significant importance, there is no standardized training for SLOE authors and CORD guidelines are infrequently reviewed by letter writers [5]. Additionally, the experience of the letter writer directly affects scores, with less experienced graders more likely to inflate scores [6]. Familiarity with a student can also play a role, with the length of time a letter writer knew an applicant significantly affecting scores [6]. As the number of emergency medicine applicants rotating through community emergency departments increases, so does the number of SLOEs from non‐residency based (nRB) physicians. This relatively new type of SLOE presents potential issues for program directors regarding its interpretation and direct comparison to the eSLOE 2.0 completed by residency based (RB) physicians.

The primary aim of this article was to compare SLOE grading between RB and nRB faculty.

## 2 Methods

### 2.1 Study Design

This study employed a multi‐institutional, cross‐sectional design to analyze eSLOE 2.0 submissions from candidates applying to five EM residency programs in the United States (both 3 and 4 years formats represented) during the 2022–2023 application period. We adhered to the Strengthening the Reporting of Observational Studies in Epidemiology guidelines [7]. This study was deemed exempt by the institutional review board at all five institutions.

### 2.2 Data Collection/Study Protocol

We obtained applicant information through the Electronic Residency Application Service (ERAS), including the Association of American Medical Colleges (AAMC) identification numbers and eSLOEs 2.0. Data collected from each eSLOE included the grade for the rotation, evaluations for sections A and B, and the Anticipated Guidance (AG) level. Trained abstractors from each institution used a pre‐tested standardized tool to gather this information, with a 10% sample re‐evaluated for consistency, achieving 98% interrater reliability. A data‐sharing agreement between the institutions permitted only the exchange of AAMC numbers. Duplicate records were removed before the final data analysis.

Data were initially compiled and anonymized in Microsoft Excel, with scores converted to a numerical scale for analysis: Section A (1–3), Section B (1–5), AG (1–4). Exclusion criteria included SLOEs not written by a faculty group, PD, associate/assistant PD, clerkship director, chair, or any combination of these; letter writer with < 5 SLOEs written the previous year; and letters not using the SLOE 2.0 format. Data were extracted from eSLOEs, including EM rotation grade, and rankings for all Evaluation of Student sections, with numerical values assigned to each ranking: Part A (fully entrustable = 3, mostly entrustable = 2, pre‐entrustable = 1), Part B (1–5), AG (minimal = 4, standard = 3, moderate = 2, most = 1). Grades were converted into a 5 point scale (honors/A = 5, high pass/B = 4, pass/C = 3, Low Pass/D = 2, E = 1, Fail = 0).

### 2.3 Data Analysis

Data were first examined to identify a suitable analysis for comparing EM RB and nRB faculty grading. In prior work, SLOE scores were analyzed using regression analyses [8, 9, 10]. In this study, all scores for Parts A, B, and AG deviated from normal. Therefore, nonparametric analyses were conducted. The chi‐squared (*χ* ^2^) statistic was calculated to compare the distribution of Part A and B ordinal scores from departments with EM residency programs to those without. Average eSLOE 2.0 scores and standard deviation (SD) were reported. eSLOEs graded by RB faculty were evaluated using Spearman's rho to measure the relationship between Grades and Part A and B scores as well as AG.

## 3 Results

A total of 1891 applicants from five residency programs were available for analysis. Exclusion criteria led to the removal of 119 applicants. A total of 1772 unique applicants (3687 total eSLOEs) were included in this analysis, representing approximately 83% of MD and DO applicants in the 2023 Match. Of these, 3526 (95.6%) were written by EM RB faculty and 161 (4.4%) by nRB faculty.

### 3.1 Part A and B Evaluations

Median scores across Parts A (clinical skills) and B (professional attributes) were similar between groups. However, score distributions differed significantly for nearly all items, as listed in Table 1. After Bonferroni correction (*α* = 0.004), statistically significant differences remained for all items except A4 (procedural skills).

**TABLE 1:** Distribution of scores for Part A, B and C questions, for eSLOEs graded by RB writers and nRB writers. We set α = 0.05; however, due to multiple comparisons, we applied a Bonferroni correction to establish α = 0.004. The proportion of each rating is indicated, rounded up.

Evaluation of student Parts A and B	EM RB writers	EM nRB writers	Chi‐squared statistic, *p*
	*N* = 3526	*N* = 161	
	Median rating (%)	Median rating (%)	
A1. Ability to perform a focused history and physical exam			
Median	3	3	*χ* ^2^ = 25.41, *p* < 0.001
Total pre‐entrustable (1)	38 (1.1%)	0 (0.0%)	
Total mostly entrustable (2)	891 (25.3%)	14 (8.7%)	
Total fully entrustable (3)	2597 (73.7%)	147 (91.3%)	
A2. Ability to generate a differential diagnosis			
Median	3	3	*χ* ^2^ = 11.76, *p* = 0.003
Total pre‐entrustable (1)	96 (2.7%)	1 (0.6%)	
Total mostly entrustable (2)	1482 (42.0%)	50 (31.1%)	
Total fully entrustable (3)	1948 (55.2%)	110 (68.3%)	
A3. Ability to formulate a plan			
Median	2	3	*χ* ^2^ = 7.32, *p* = 0.03
Total pre‐entrustable (1)	119 (3.4%)	1 (0.6%)	
Total mostly entrustable (2)	1749 (49.6%)	70 (43.5%)	
Total fully entrustable (3)	1658 (47.0%)	90 (55.9%)	
A4. Ability to perform common ED procedures			
Median	3	3	*χ* ^2^ = 0.15, *p* = 0.99
Not applicable (0)	216 (6.1%)	9 (5.6%)	
Total pre‐entrustable (1)	53 (1.5%)	2 (1.2%)	
Total mostly entrustable (2)	1430 (40.6%)	66 (41.0%)	
Total fully entrustable (3)	1827 (51.8%)	84 (52.2%)	
A5. Ability to recognize and manage basic emergent situations			
Median	3	3	*χ* ^2^ = 7.17, *p* = 0.03
Total pre‐entrustable (1)	78 (2.2%)	1 (0.6%)	
Total mostly entrustable (2)	1345 (38.1%)	48 (29.8%)	
Total fully entrustable (3)	2103 (59.6%)	112 (69.6%)	
B1. Compassion, sensitivity, and respect towards patients and team members			
Median	4	5	*χ* ^2^ = 32.84, *p* < 0.001
Total minimally acceptable for an EM resident (1)	2 (0.1%)	0 (0.0%)	
Total (2)	22 (0.6%)	1 (0.6%)	
Total (3)	471 (13.4%)	11 (6.8%)	
Total (4)	1430 (40.6%)	39 (24.2%)	
Total exceptional EM candidate (5)	1601 (45.4%)	110 (68.3%)	
B2. Receptivity to feedback and ability to incorporate feedback			
Median	4	5	*χ* ^2^ = 22.36, *p* < 0.001
Not‐applicable (0)	1 (0.0%)	0 (0.0%)	
Total minimally acceptable for an EM resident (1)	2 (0.1%)	0 (0.0%)	
Total (2)	48 (1.4%)	1 (0.6%)	
Total (3)	497 (14.1%)	7 (4.3%)	
Total (4)	1477 (41.9%)	58 (36.0%)	
Total exceptional EM candidate (5)	1501 (42.6%)	95 (59.0%)	
B3. Dependability, responsibility, initiative, and work ethic			
Median	4	5	*χ* ^2^ = 31.45, *p* < 0.001
Not applicable (0)	1 (0.0%)	0 (0.0%)	
Total minimally acceptable for an EM resident (1)	4 (0.1%)	0 (0.0%)	
Total (2)	45 (1.3%)	0 (0.0%)	
Total (3)	453 (12.8%)	6 (3.7%)	
Total (4)	1278 (36.2%)	41 (25.5%)	
Total exceptional EM candidate (5)	1745 (49.5%)	114 (70.8%)	
B4. Punctuality, attendance, and preparation for duty			
Median	4	5	*χ* ^2^ = 25.09, *p* < 0.001
Not‐applicable (0)	1 (0.0%)	0 (0.0%)	
Total minimally acceptable for an EM resident (1)	4 (0.1%)	0 (0.0%)	
Total (2)	34 (1.0%)	0 (0.0%)	
Total (3)	479 (13.6%)	7 (4.3%)	
Total (4)	1272 (36.1%)	45 (28.0%)	
Total exceptional EM candidate (5)	1736 (49.2%)	109 (67.7%)	
B5. Timeliness and responsiveness in completing administrative tasks			
Median	4	5	*χ* ^2^ = 37.26, *p* < 0.001
Not‐applicable (0)	1 (0.0%)	2 (1.2%)	
Total minimally acceptable for an EM resident (1)	3 (0.1%)	0 (0.0%)	
Total (2)	54 (1.5%)	1 (0.6%)	
Total (3)	574 (16.3%)	15 (9.3%)	
Total (4)	1335 (37.9%)	56 (34.8%)	
Total exceptional EM candidate (5)	1559 (44.2%)	87 (54.0%)	
B6. Interpersonal and communication skills with patients and family members			
Median	4	5	*χ* ^2^ = 23.31, *p* < 0.001
Total minimally acceptable for an EM resident (1)	2 (0.1%)	0 (0.0%)	
Total (2)	26 (0.7%)	0 (0.0%)	
Total (3)	461 (13.1%)	10 (6.2%)	
Total (4)	1441 (40.9%)	48 (29.8%)	
Total exceptional EM candidate (5)	1596 (45.3%)	103 (64.0%)	
B7. Interpersonal and communication skills with faculty, residents and healthcare professionals			
Median	4	5	*χ* ^2^ = 23.24, *p* < 0.001
Total minimally acceptable for an EM resident (1)	12 (0.3%)	0 (0.0%)	
Total (2)	68 (1.9%)	1 (0.6%)	
Total (3)	523 (14.8%)	7 (4.3%)	
Total (4)	1327 (37.6%)	54 (33.5%)	
Total exceptional EM candidate (5)	1596 (45.3%)	99 (61.5%)	
C1. Anticipated guidance			
Median	3	4	*χ* ^2^ = 29.66, *p* < 0.001
Most (1)	87 (2.5%)	3 (1.9%)	
Moderate (2)	424 (12.0%)	2 (1.3%)	
Standard (3)	1815 (51.5%)	73 (45.6%)	
Minimal (4)	1200 (34.0%)	82 (51.2%)	

In Part A, nRB faculty consistently assigned fewer ratings of “1” and “2” and more ratings of “3” (fully entrustable). For example, 91% of nRB group rated students as fully entrustable in history‐taking (A1), compared to 74% in the RB group.

In Part B, nRB faculty were more likely to give the highest rating (“5”) across professionalism domains. For B3 (work ethic), 71% of nRB faculty selected “5” compared with 50% of RB faculty. This pattern held across B1–B7.

### 3.2 Part C: Anticipated Guidance

The most frequent score for Part C (anticipated level of guidance) differed between groups. nRB faculty more often selected the highest AG score (“4”), indicating less anticipated guidance (51% vs. 34%; *χ* ^2^ = 29.66, *p* < 0.001).

### 3.3 Correlations With Final Grade and Anticipated Guidance

Correlations between individual item scores and the final grade and correlations between individual scores and AG are presented in Table 2. Among RB faculty, both Parts A and B correlated moderately with the final grade, highest for A1 and B3 (*ρ* = 0.32 each). For nRB faculty, correlations with the final grade were generally lower for Part A (particularly in items A4 and A5) and slightly higher for Part B, suggesting professionalism ratings may influence grades more in this group.

**TABLE 2:** Correlations of Parts A and B scores with Grade and Part C for all eSLOEs graded by RB r and nRB faculty (*N* = 3526). We set α = 0.05; however, due to the multiple comparisons, we applied a Bonferroni correction to establish α = 0.004.

Evaluation of student	Grade		C1 anticipated guidance	
	RB writers	nRB writers	RB writers	nRB writers
Parts A and B	*N* = 3526	*N* = 161	*N* = 3526	*N* = 161
A1. Ability to perform a focused history and physical exam. Fully entrustable = 3 Mostly entrustable = 2 Pre‐entrustable = 1	*⍴* = 0.32, *p* < 0.001	*⍴* = 0.25, *p* = 0.003	*⍴* = 0.48, *p* < 0.001	*⍴* = 0.27, *p* < 0.001
A2. Ability to generate a differential diagnosis. Fully entrustable = 3 Mostly entrustable = 2 Pre‐entrustable = 1	*⍴* = 0.29, *p* < 0.001	*⍴* = 0.17, *p* = 0.05	*⍴* = 0.51, *p* < 0.001	*⍴* = 0.41, *p* < 0.001
A3. Ability to formulate a plan. Fully entrustable = 3 Mostly entrustable = 2 Pre‐entrustable = 1	*⍴* = 0.30, *p* < 0.001	*⍴* = 0.20, *p* = 0.02	*⍴* = 0.53, *p* < 0.001	*⍴* = 0.35, *p* < 0.001
A4. Ability to perform common ED procedures. Fully entrustable = 3 Mostly entrustable = 2 Pre‐entrustable = 1, N/A = 0	*⍴* = 0.15, *p* < 0.001	*⍴* = 0.05, *p* = 0.56	*⍴* = 0.34, *p* < 0.001	*⍴* = 0.41, *p* < 0.001
A5. Ability to recognize and manage basic emergent situations. Fully entrustable = 3 Mostly entrustable = 2 Pre‐entrustable = 1	*⍴* = 0.29, *p* < 0.001	*⍴* = 0.04, *p* = 0.61	*⍴* = 0.47, *p* < 0.001	*⍴* = 0.28, *p* < 0.001
B1. Compassion, sensitivity, and respect towards patients and team members. 5, 4, 3, 2, 1 (5 = exceptional EM candidate, 1 = minimally acceptable for an EM resident)	*⍴* = 0.25, *p* < 0.001	*⍴* = 0.36, *p* < 0.001	*⍴* = 0.45, *p* < 0.001	*⍴* = 0.39, *p* < 0.001
B2. Receptivity to feedback and ability to incorporate feedback. 5, 4, 3, 2, 1 (as above in B1)	*⍴* = 0.27, *p* < 0.001	*⍴* = 0.43, *p* < 0.001	*⍴* = 0.50, *p* < 0.001	*⍴* = 0.34, *p* < 0.001
B3. Dependability, responsibility, initiative, and work ethic. 5, 4, 3, 2, 1 (as above in B1)	*⍴* = 0.32, *p* < 0.001	*⍴* = 0.40, *p* < 0.001	*⍴* = 0.56, *p* < 0.001	*⍴* = 0.33, *p* < 0.001
B4. Punctuality, attendance, and preparation for duty. 5, 4, 3, 2, 1 (as above in B1)	*⍴* = 0.22, *p* < 0.001	*⍴* = 0.25, *p* = 0.003	*⍴* = 0.45, *p* < 0.001	*⍴* = 0.34, *p* < 0.001
B5. Timeliness and responsiveness in completing administrative tasks. 5, 4, 3, 2, 1 (as above in B1)	*⍴* = 0.20, *p* < 0.001	*⍴* = 0.26, *p* = 0.002	*⍴* = 0.45, *p* < 0.001	*⍴* = 0.36, *p* < 0.001
B6. Interpersonal and communication skills with patients and family members. 5, 4, 3, 2, 1 (as above in B1)	*⍴* = 0.30, *p* < 0.001	*⍴* = 0.31, *p* < 0.001	*⍴* = 0.53, *p* < 0.001	*⍴* = 0.31, *p* < 0.001
B7. Interpersonal and communication skills with faculty, residents and healthcare professionals. 5, 4, 3, 2, 1 (as above in B1)	*⍴* = 0.32, *p* < 0.001	*⍴* = 0.37, *p* < 0.001	*⍴* = 0.56, *p* < 0.001	*⍴* = 0.38, *p* < 0.001

In contrast, Part A and B scores were more strongly correlated with the AG rating (Part C), especially among RB faculty. The highest correlations were seen for B3 (*ρ* = 0.56) and A3 (*ρ* = 0.53). While similar trends were observed in the nRB group, the correlations were generally lower and more variable.

## 4 Discussion

As medical schools expand and students rotate in departments without residency programs, it is essential to consider the source of evaluation data when selecting applicants. This study found significant grading differences between RB and nRB faculty on most Part A and B items and the Part C AG question of the SLOE, with nRB faculty more frequently assigned higher scores.

The question of *why* differences exist is beyond the scope of this paper as we did not assess the characteristics of the study populations. We did not match for differences in student background (MD, DO, international) rotating at the programs. Additionally, we do not have insight into the level of experience of the letter writers providing SLOEs. These variables may play a role in the score distribution; however, there is a paucity of research to further inform these possibilities.

Several prior studies speak to how programs interpret SLOEs in ranking decisions. Love et al. [3] found that SLOEs are heavily weighted for interview offers, but “inflated evaluations” were the leading cause of diminished value. A qualitative paper by Schrepel et al. [11] discusses that “contextualizing” the SLOE in terms of letter writer, institution, and prior patterns of SLOE writing played a role in the interpretation of the SLOE and in estimates of student competitiveness. Awareness of the scoring trends in this study may help programs decide how much weight to assign each SLOE type. These differences support a holistic application review and careful consideration when using numerical scores for screening. Faculty reviewers should be trained in best practices for interpreting SLOEs.

This paper did not assess narrative themes of the written portion of the SLOE but would be an interesting future direction and may provide additional insight into differences between different SLOE types. Additional future directions include assessing the letter writer experience/position and their relation to grading patterns as well as comparing individuals who had both nRB and RB letters and accounting for maturation effects.

## 5 Limitations

Our data set is limited to select EM programs, and although our data included 88.5% of all applicants in the 2023 Match, it does not reflect all SLOEs. The data were also limited by the small sample size of the nRB group as compared to the RB group. As this study was not designed to identify causal factors, we are unable to determine the reason for the identified differences, and it is possible that there are confounding factors beyond the credentials of the letter writer. Additionally, our analysis of SLOE Grades is limited by various grading scales used by programs; while we created a standardized scale to align grades along an ordinal scale, it may not fully reflect the intricacies of grading systems. Finally, this study is limited to the RB and nRB SLOE 2.0 and may not apply to other versions of the SLOE such as the sub‐specialty and off‐service SLOE formats.

## 6 Conclusion

When assessing SLOEs 2.0, this study identified that a larger proportion of higher end scores were from nRB faculty when compared to those from traditional RB programs.

## Author Contributions

**Katarzyna Gore:** conceptualization, data curation, formal analysis, methodology, supervision, writing – review and editing, writing – original draft. **Cullen B. Hegarty:** data curation, writing – original draft, writing – review and editing. **Thomas Beardsley:** data curation, writing – original draft, writing – review and editing. **Sara M. Krzyzaniak:** data curation, writing – original draft, writing – review and editing. **Sandra Monteiro:** conceptualization, formal analyis, methodology, writing – original draft, writing – review and editing. **Al'ai Alvarez:** conceptualization, data curation, methodology, writing – review and editing. **Teresa Davis:** data curation, writing – review and editing. **Melissa Parsons:** data curation, writing – review and editing. **Aman Pandey:** data curation, writing – review and editing. **Sharon Bord:** data curation, writing – review and editing. **Michael Gottlieb:** conceptualization, data curation, formal analysis, methodology, supervision, writing – original draft, writing – review and editing. **Alexandra Mannix:** conceptualization, data curation, formal analysis, methodology, supervision, writing – original draft, writing – review and editing.

## Funding

The authors have nothing to report.

## Conflicts of Interest

The authors declare no conflicts of interest.

## Data Availability

The data that support the findings of this study are available on request from the corresponding author. The data are not publicly available due to privacy or ethical restrictions.
